# Digital plasmonic holography

**DOI:** 10.1038/s41377-018-0049-2

**Published:** 2018-08-15

**Authors:** Joseph W. Nelson, Greta R. Knefelkamp, Alexandre G. Brolo, Nathan C. Lindquist

**Affiliations:** 1 Department of Physics and Engineering, Bethel University, 3900 Bethel Drive, St Paul, MN 55112 USA; 2 Department of Chemistry, University of Victoria, 3800 Finnerty Road, Victoria, BC V8P 5C2 Canada; 3 Center for Advanced Materials and Related Technologies (CAMTEC), University of Victoria, 3800 Finnerty Road, Victoria, BC V8P 5C2 Canada

## Abstract

We demonstrate digital plasmonic holography for direct in-plane imaging with propagating surface-plasmon waves. Imaging with surface plasmons suffers from the lack of simple in-plane lenses and mirrors. Lens-less digital holography techniques, however, rely on digitally decoding an interference pattern between a reference wave and an object wave. With far-field diffractive optics, this decoding scheme provides a full recording, i.e., a hologram, of the amplitude and phase of the object wave, giving three-dimensional information from a two-dimensional recording. For plasmonics, only a one-dimensional recording is needed, and both the phase and amplitude of the propagating plasmons can be extracted for high-resolution in-plane imaging. Here, we demonstrate lens-less, point-source digital plasmonic holography using two methods to record the plasmonic holograms: a dual-probe near-field scanning optical microscope and lithographically defined circular fluorescent screens. The point-source geometry gives in-plane magnification, allowing for high-resolution imaging with relatively lower-resolution microscope objectives. These results pave the way for a new form of in-plane plasmonic imaging, gathering the full complex wave, without the need for plasmonic mirrors or lenses.

## Surface plasmons: Digital tricks generate real-time holograms without lenses

Researchers have exploited the extreme sensitivity of plasmon waves toward surface features for a holographic imaging system fast enough to monitor microsecond-scale binding processes. The approach from Nathan Lindquist at Bethel University in the U.S.A. and colleagues at the University of Victoria in Canada uses laser excitation to direct coherent plasmon waves towards objects such as microbeads located on ultra-smooth silver films. The interference patterns between the reference waves and ones emanating from the sample formed distinct holograms, captured with a scanning probe tip or fluorescent film. Digital reconstruction techniques enabled the team to reproduce the plasmonic field without plasmonic optical elements and produce high-resolution holograms of multiple objects. Because this technique can generate holograms from a single one-dimensional scan — existing plasmonic sensors need slower two-dimensional data — it may find use in real-time imaging applications.

## Introduction

The interdisciplinary fields of plasmonics and nanophotonics allow manipulation of light with sub-wavelength precision. Surface plasmons (SPs) are electromagnetic surface waves bound to a metal-dielectric interface by coupling to free electrons^1^. SPs have a shorter wavelength than free-space light and extend only ~100 nm into their surrounding environment. As they propagate, plasmons can scatter from surface defects, diffract around nanostructures, and cause interference effects with incident light, re-radiated light, or even other plasmons^2^. Because of these near-field effects, plasmons have been explored for many applications, including photovoltaics, enhanced spectroscopy, and sensing^3,4^. To better control these surface waves, there has been significant interest in developing the two-dimensional equivalents of standard optical elements, including mirrors, beam-splitters, and interferometers^5^ and in studying in-plane plasmon diffraction^6^, refraction^7^, or wavefront shaping^8^. In-plane and out-of-plane plasmonic lenses have been demonstrated^9–12^ but rely on complex fabrication techniques^13^ and have not been fully developed for imaging purposes. Using other plasmonic optical elements, direct in-plane imaging has also been explored^14^. For example, parabolic plasmonic mirrors^15^ will form an in-plane image but again rely on complex fabrication of the nanostructures. While leakage radiation microscopy can image the intensity of plasmons at a surface^16^, it relies on energy loss through a thin metal film in the region of interest and very high numerical aperture (NA) optics. Direct imaging of plasmon waves over a wide area on an optically thick, smooth, and otherwise unperturbed metallic surface would offer many advantages. For example, since plasmon waves have been used extensively for biosensing^17^, directly visualizing their interactions—i.e., how they diffract, refract, and scatter from various objects—could aid in optimizing and characterizing novel sensing approaches.

One solution to the lack of simple in-plane plasmonic imaging elements is to consider an imaging approach that does not rely on lenses or mirrors at all. In particular, the emergence of digital techniques has revolutionized far-field imaging through digital holographic microscopy^18–25^ (DHM). These digital techniques explicitly reproduce the propagation of light waves. Because all recording media (e.g., a charge-coupled device (CCD) camera) respond only to the intensity of light, gathering phase information requires a mutually coherent reference wave to interfere with the object wave, forming a hologram. When this hologram is recorded on a digital imager, even without a lens, the known reference wave can be used to extract the phase and amplitude of the unknown object wave. The object wave can then be propagated digitally through space. A two-dimensional recorded hologram can therefore be used to determine the object wave at any point in three dimensions. Recording the full complex wave in this way offers many advantages such as the freedom to choose any imaging modality, e.g., replicating differential interference contrast microscopy^26,27^, even after the data have been gathered. Point-source, lens-less DHM in particular^28^ has shown significant promise for extremely simple, high-resolution, real-time imaging of particles, cells, surfaces, and other microscopic objects^29^. Along with other lens-less imaging techniques^30^, DHM has significant potential for biological imaging and sensing^31^. For example, DHM has been used previously to image microorganisms^32^ and to track cells^33^.

Unfortunately, these techniques have not yet been fully realized in near-field optics. While digital techniques have been used to study the fields beamed from plasmonic apertures^34^, to recover diffraction patterns for sensing^35,36^, or to image prism-coupled plasmons^37^, in-plane imaging and explicitly modeling the two-dimensional diffraction and propagation of the plasmons over a metallic surface were not fully explored. Plasmonic imaging with a digital scanning element has also been demonstrated^38^, but like all intensity-only plasmonic imaging techniques^14–16^, did not give the benefits of holography described above to record the full complex wave. Finally, while Fourier plasmon optics has also been investigated^39,40^, as has holographic excitation of plasmon waves for plasmonic tweezing applications^41^, these techniques have not been used for imaging.

Holography is a well established and powerful optical technique, as are its more recent digital implementations that have enjoyed many important applications. Therefore, much is to be gained in exploring these techniques in plasmonics. For example, the refractive index sensitivity and imaging capabilities of holography could further the development of multiplex plasmonic biosensing. Directly visualizing surface-plasmon waves may also facilitate fundamental studies of plasmon/nanoparticle interactions. Therefore, to enable these new areas of research, in this paper we demonstrate the plasmonic equivalent of lens-free, point-source, in-line DHM for high-resolution in-plane imaging. In this case, a one-dimensional plasmonic hologram is used to reconstruct an image of the two-dimensional surface through which the plasmons are propagating.

## Results

**Fig. 1:**
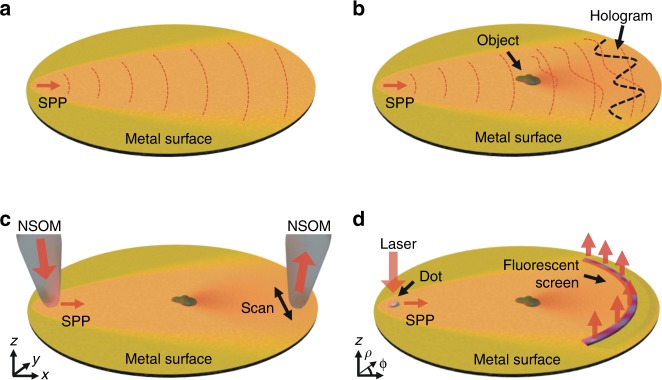
Experimental concept. **a** A plasmon reference wave propagates over a surface and **b** interacts with micro-objects forming the scattered object wave. The object wave interferes with the reference wave forming a plasmonic hologram. The hologram is accessed via **c** a dual-probe NSOM system or with **d** a fluorescent screen. The hologram was typically recorded in the *y* or *ϕ* direction and after the known reference wave is removed, the field can be back-propagated in the -*x* or *-ρ* direction to reconstruct the amplitude and phase of the original object wave

Figure 1 shows the experimental scheme for plasmonic DHM wherein the interference between a reference plasmon wave and an object plasmon wave forms a hologram on a smooth metallic surface. A point-source plasmon wave propagates across a smooth metallic surface (Fig. 1a) and interacts with objects on the surface (Fig. 1b). The expanding reference wave $R(x,y)$ and the scattered object wave $O(x,y)$ interfere as a hologram $H(x,y)$:

1 $$H\left( {x,y} \right) = R\left( {x,y} \right) + O\left( {x,y} \right)$$

This holographic pattern was accessed in two ways: directly with a dual-probe near-field scanning optical microscope (NSOM, Nanonics Multiview^TM^ 4000) and indirectly by imaging the plasmon interaction with a dye-doped (Oxazine 750) fluorescent film of patterned polymethylmethacrylate (PMMA) electron-beam resist. Hankel-type plasmons^42^ are emitted from single nanoapertures (Fig. 1c) or nanobumps (Fig. 1d) and provide the point-source reference wave ${R}\left(x,y \right)$ in our experiments. The two experiments had two different laser excitation sources. The wavelength of the surface plasmon was calculated from dielectric constants in published experimental data^43^. The NSOM experiments used a 638 nm laser, giving a plasmon wavelength of $\lambda _{\mathrm {SP}} = 620\,{\mathrm{nm}}$. The fluorescent PMMA screen experiments used a 660 nm laser, giving a plasmon wavelength of $\lambda _{\mathrm {SP}} = 640\,{\mathrm{nm}}$. The plasmon wavenumber is defined as $k_{\mathrm {SP}} = \frac{{2\pi }}{{\lambda _{\mathrm {SP}}}}$. The recording media (the second NSOM probe in Fig. 1c or the PMMA screen in Fig. 1d) are sensitive only to the hologram intensity $\left| {H\left( {x,y} \right)} \right|^2$ as follows:

2 $$\left| {H\left( {x,y} \right)} \right|^2 = \left| {R\left( {x,y} \right)} \right|^2 \; + \; \left| {O\left( {x,y} \right)} \right|^2 \; + \; R^ \ast \left( {x,y} \right)O\left( {x,y} \right) \\ \; + \; R\left( {x,y} \right)O^ \ast \left( {x,y} \right)$$

Typically, with the small particle scattering centers as used in this work, $\left| {O\left( {x,y} \right)} \right|^2$ is much smaller than $\left| {R\left( {x,y} \right)} \right|^2$ and is ignored. The slowly varying reference term $\left| {R\left( {x,y} \right)} \right|^2$ can then be removed with a digital high-pass filter, leaving only the two cross terms. Finally, multiplying by a digital copy of the known reference wave $R(x,y)$ leaves only the object wave $O(x,y)$ and its so-called twin image^18^. These terms, referred to as the hologram $E^{SP}(x = a,y)$ henceforth, were recorded with the NSOM in the $y$ direction at the location $x = a$ or with the fluorescent screen in the *ϕ* direction at the location $\rho = a$. The hologram can then be propagated digitally to any other point in the plane using various wave propagation algorithms. While the twin image can provide significant interference, especially with the short propagation and recording distances inherent in plasmonic DHM shown here, several solutions are available and are discussed further below.

The plasmonic equivalent^44^ of the angular spectrum technique^18^ was used to back-propagate the *z* component of the plasmonic field $E^{\mathrm {SP}}(x = a,y)$ at the surface from $x = a$ towards $x = 0$, the location of the source. While the plasmonic field is vectorial and polarization effects should sometimes be considered^44^, such a scalar approximation is sufficient for field propagation and other field components can still be derived from the *z* component. As shown by various researchers^39,44^, the plasmonic field $E^{\mathrm {SP}}(x,y)$ propagating from $x = a$ in the −*x* direction can be solved via a Fourier transform operation as follows:

3 $$E^{\mathrm {SP}}(x,y) = \frac{1}{{2\pi }}{\int} {E^{\mathrm {SP}}(x = a;k_y)e^{ - i\sqrt {k_{\mathrm {SP}}^2 - k_y^2} (x - a)}e^{ik_yy}dk_y}$$

Here, $E^{\mathrm{SP}}(x = a;k_y)$ is the angular spectrum (i.e., the Fourier transform) of the hologram at the location $x = a$ after the background terms have been removed as described above. The transfer function from $x = a$ to another location $x$ is given by $e^{ - i\sqrt {k_{\mathrm {SP}}^2 - k_y^2} (x - a)}$, where $k_{SP}^2 = k_x^2 + k_y^2$. The in-plane wavenumber $k_{\mathrm {SP}}$ also has an imaginary component that represents the material losses inherent in plasmon propagation. In our case, the hologram recording distances (~25 µm) were smaller than the plasmon propagation length for red light on silver (~60 µm). The propagation losses therefore did not significantly impact plasmonic image formation. This lack of impact would not have been the case for larger recording distances or for other wavelengths and materials^44^. The out-of-plane *z* dependence of the field as well as the $e^{i\omega t}$ time dependence have both been omitted in the above equations for brevity.

In some experiments, a circular recording geometry was used to match the symmetry of the point-source plasmon reference wave as it propagates radially, as shown in Fig. 1d. The following is completely analogous to the angular spectrum method^45^ and is applied here to the propagation of plasmons on a surface. In this case, the plasmonic field $E^{\mathrm {SP}}(\rho ,\phi )$ is given in polar coordinates and is propagated radially inward with $\rho < a$ using the Fourier series relationship given here:

4 $$E^{\mathrm {SP}}(\rho ,\phi ) = \mathop {\sum }\limits_{n = - \infty }^{n = \infty } E_n^{\mathrm {SP}}(\rho = a)\frac{{H_n^{(2)}\left( {k_{\mathrm {SP}}a} \right)}}{{H_n^{(2)}(k_{\mathrm {SP}}\rho )}}e^{in\phi }$$

In this case, $H_n^{\left( 2 \right)}$ are Hankel functions of the second kind defining the transfer function $\frac{{H_n^{(2)}(k_{SP}a)}}{{H_n^{(2)}(k_{SP}\rho )}}$, whereas $E_n^{\mathrm {SP}}(\rho = a)$ is the transform in polar coordinates of the hologram recorded at $\rho = a$, again after removing the background terms, given as:

5 $$E_n^{\mathrm {SP}}(\rho = a) = \frac{1}{{2\pi}} {\int}_0^{2\pi} E^{\mathrm {SP}}(\rho = a,\phi )e^{ - in\phi}d\phi$$

and is sometimes called the helical wave spectrum^45^. Aside from the change of coordinates and the two different transfer functions, the methods given in Eqs. (3) and (4) are completely analogous^45^ and can both be used to propagate the plasmonic fields from the hologram location to the object position, depending on the recording geometry.

**Fig. 2:**
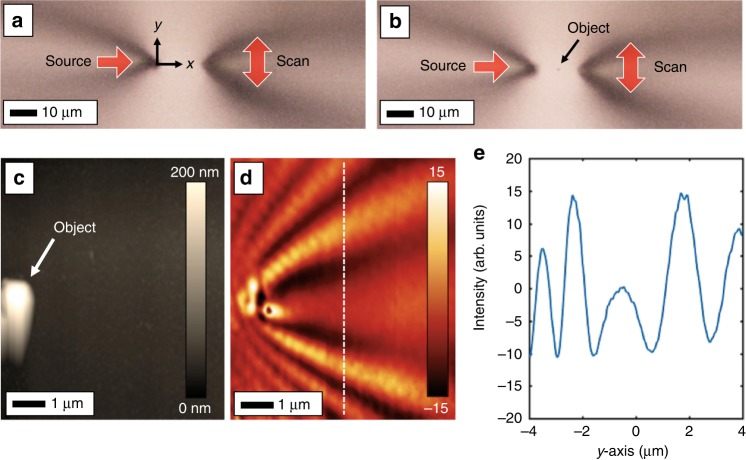
Plasmonic DHM proof-of-concept with a dual-probe NSOM. **a** The two-probe setup with a stationary source and second scanning probe to record the hologram. The recording axes and geometry are shown. **b** A polystyrene sphere is placed between the two probes by moving the sample stage. **c** AFM scan of the object. **d** Simultaneous NSOM image of plasmons scattering from the object and interfering with the source wave. The background offset has been removed. The dashed vertical line shows (**e**) a cross-section of the hologram

Figure 2 shows scans with the dual-probe NSOM setup. The first probe was the source, and the second probe scanned the surface laterally in the *y* direction. The objects were randomly scattered polystyrene beads. Figure 2a shows a microscope image of the dual-probe setup, and Fig. 2b shows the insertion of a single bead object by moving the sample stage. The NSOM system also provides height feedback in the form of an atomic force microscope (AFM) scan (Fig. 2c). The concurrent NSOM image (Fig. 2d) shows plasmon waves diffracting around the object and forming a standing wave pattern beyond the object. After the background is removed as described above, a cross-section is taken laterally in the *y* direction, showing the holographic pattern (Fig. 2e).

**Fig. 3:**
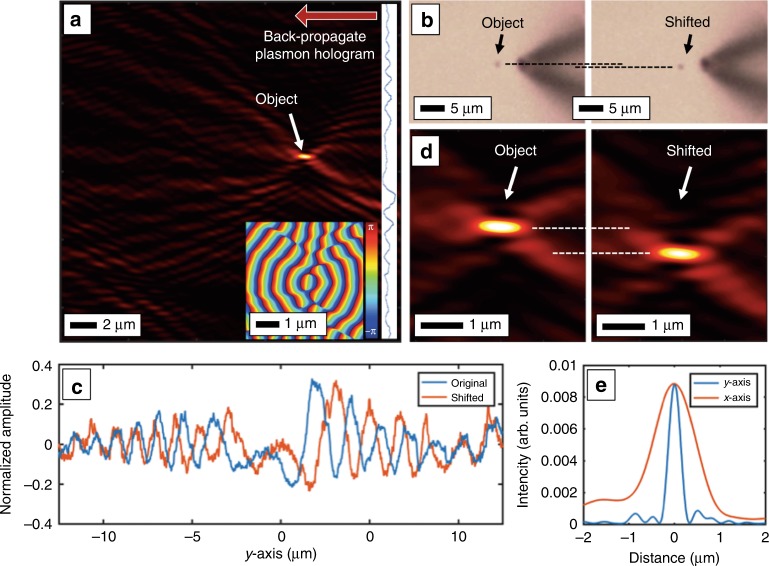
Plasmonic DHM of a single-point object. **a** A digital reconstruction of a plasmonic hologram of a single polystyrene sphere. The hologram data are shown vertically where they were recorded, and the reconstruction shows the field intensity, giving the location of the original object. (inset) The phase of the reconstruction shows the converging plasmon wavefront. **b** Two successive holograms were taken of the same, but laterally shifted, object. **c** This object shift appears as a shift in the hologram. **d** The reconstruction of the two mutually shifted holograms reconstructs the object at the correct locations. **e** A cross-section of the reconstruction in the *x* and *y* directions

While a full-area scan with the NSOM of the object provides the plasmonic field intensities in the plane, a single-line scan at some distance, i.e., the dashed line in Fig. 2d, will provide the one-dimensional hologram. Numerical techniques can then be used to back-propagate the plasmonic field one step at a time, forming a two-dimensional image. Figure 3 shows plasmonic DHM of the single microspherical object. In this case, the second NSOM probe was scanned at 25 µm from the source with the object placed between the two probes as before. The scan covered 25 µm in the *y* direction, thereby defining an ~25 µm by 25 µm imaging area. The hologram was back-propagated using Eq. (3) to form an image of the object (Fig. 3a). The digital propagation also contains phase information for the plasmonic field, shown in the inset. To test the capabilities of plasmonic DHM, the object was shifted 1 µm in the *y* direction using the sample stage (Fig. 3b), shifting the recorded hologram (Fig. 3c) as well as the reconstructed image (Fig. 3d) and showing high-resolution capabilities. Figure 3e shows a cross-section of the reconstruction. The test object in this case was a ~200 nm diameter sphere, smaller than the plasmon wavelength. The reconstruction is therefore a plasmon-diffraction-limited image of the point-like test object. In this case, the measured width of the reconstructed point is ~300 nm.

**Fig. 4:**
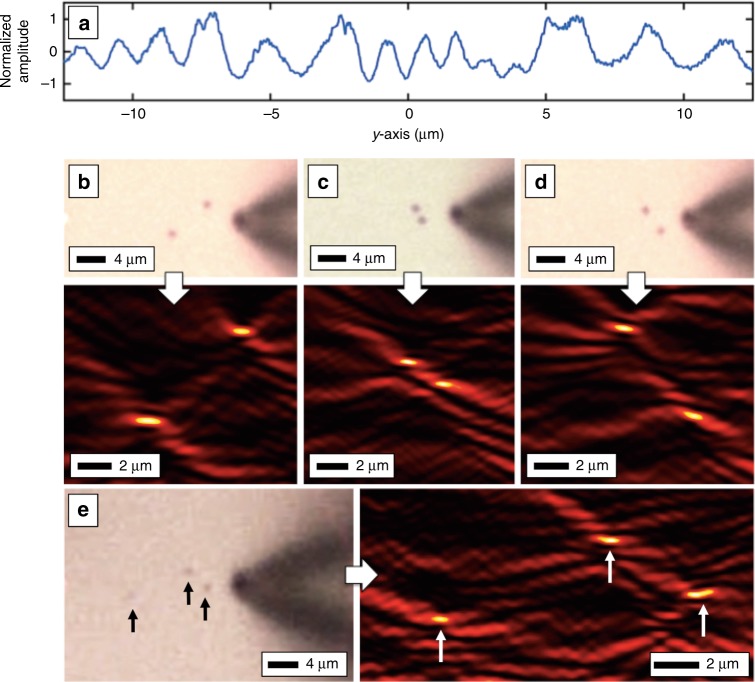
Plasmonic DHM with various point objects. **a** Representative hologram taken from an arrangement of multiple polystyrene microsphere objects. **b** A microscope image of the recording probe and two microspheres. The reconstruction correctly locates the objects, showing that the one-dimensional hologram encodes depth or distance information in two dimensions over the entire area. **c** Two closely spaced microspheres and the reconstruction. **d** Another arrangement of two microspheres and the reconstruction. **e** An arrangement of three microsphere objects and the reconstruction

Beyond imaging a single object, Fig. 4 shows that plasmonic DHM is also capable of imaging multiple objects simultaneously. In this case, the test objects were ~400 nm diameter spheres. Here, the recorded holograms look more complex (Fig. 4a), and the various arrangements of two or three objects are faithfully reconstructed (Fig. 4b–e). Plasmonic DHM is capable of imaging multiple objects over a two-dimensional area with just a single one-dimensional scan, analogous to standard DHM being capable of imaging a full three-dimensional volume with a single two-dimensional holographic recording. In one sense, unless the near-field resolution of the NSOM is needed at the object itself, plasmonic DHM drastically reduces the image acquisition time.

**Fig. 5:**
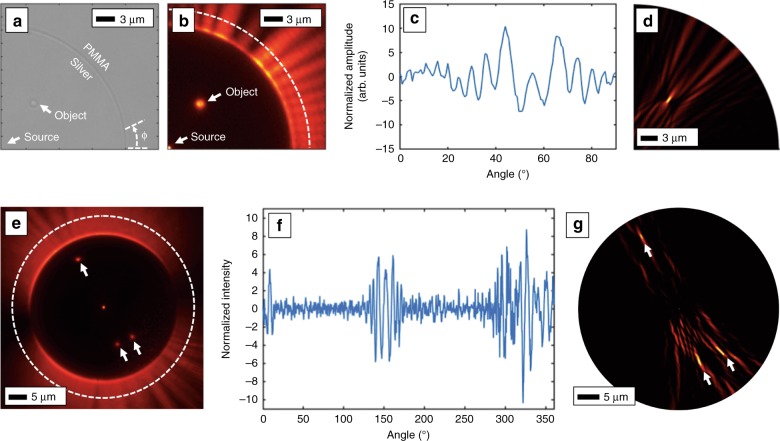
Plasmonic DHM using a fluorescent screen. **a** SEM image of a dye-doped PMMA screen to image the plasmonic hologram. The laser illuminates a small dot at the origin, producing the plasmon source. An object was also fabricated in place. **b** Fluorescence image of the plasmonic hologram. The open circle is smooth silver while the fluorescent PMMA circle provides a direct visualization of the hologram. **c** Extracting the intensities from the image along the dashed line provides the one-dimensional plasmonic hologram. **d** Digital reconstruction of the object. **e** Fluorescence image of a full circle containing three objects. The laser was polarized vertically. **f** Extracted hologram of three objects. **g** The digital reconstruction showing the three objects

Figure 5 shows plasmonic DHM with the fluorescent PMMA screen detection. An open circle of smooth silver with a central scattering dot allowed a laser beam focused in the center of the circle to produce the point-source reference plasmon wave. Instead of randomly located polystyrene spheres, the objects in this case were nanofabricated in place. Figure 5a shows an optical microscope bright-field image of a fabricated structure. The source is in the center of the 25 µm radius circle and a single test object is located approximately 10 µm away. The structure covered the full circle, but only the portion with the test object is shown here. When the laser is focused on the central dot, the fluorescent PMMA screen reveals the plasmonic hologram (Fig. 5b). Since the central dot and test object were fabricated with the same fluorescent PMMA as the circle, they also appear in the fluorescence microscope image. A circumferential profile was then extracted directly from the image and high-pass filtered to remove the background, giving a final hologram, shown in Fig. 5c. Due to the symmetry, the angular spectrum technique was modified to propagate cylindrical waves as shown in Eq. (4). The reconstruction is given in Fig. 5d, again reproducing the correct location of the point-like test object. Figure 5e shows a fluorescence image of a full circle with three test objects and the central source. The hologram was again extracted and filtered (Fig. 5f). As shown before with the NSOM scans, Fig. 5g shows that these objects are faithfully reconstructed with the reconstruction algorithms, this time using a fluorescent PMMA screen. Recording the holograms with these two separate and distinct techniques points to the versatility of plasmonic DHM.

## Discussion

The transverse resolution, magnification, effective NA, and axial resolution in a standard DHM experiment are all related directly to the wavelength and to the recording geometry, including the pixel density of the recording camera^18^. These considerations are also important in plasmonic DHM. For example, the linear NSOM scans can capture a specific range of scattering angles, depending on the recording geometry. For a scan of length *S* and an object distance of *D* from the center of the scan line, the maximum recorded scattering angle $\phi _{max}$ is $\sin \left( {\phi _{max}} \right) = \frac{{S/2}}{{\sqrt {\left( {S/2} \right)^2 + D^2} }},$ corresponding to a plasmon-diffraction-limited transverse resolution $\Delta y$ of:

6 $$\Delta y = \frac{{\lambda _{SP}}}{{2\sin \phi _{max}}}$$

and defining an effective NA $= \sin \phi _{max}$ that is analogous to the resolution criteria for standard in-line DHM^18^ and represents the rough transverse extent of the point-spread function. Refer to the Supplementary Information for a derivation of Eq. (6) as well as supplementary Figure S1 for a schematic of the recording geometries. This resolution is observed experimentally, for example, in Fig. 3a. The point-like object is a distance *D* *=* 7.0 µm from the scanned NSOM “screen” that is *S* = 25.0 µm long, corresponding to NA = 0.87, with a plasmon wavelength of $\lambda _{\mathrm {SP}} = 620$ nm and a theoretical transverse spot size of $\Delta y$ = 355 nm. Indeed, the 1/*e* full width of the object in Fig. 3e is measured to be 360 nm. Equation (6) also shows that the resolution of the reconstructed image depends on the object-to-screen distance. For example, if the distance from the object to the screen doubled to *D* *=* 14.0 µm, the plasmon-diffraction-limited resolution would be 470 nm. However, the circular fluorescent PMMA screens are able to capture all forward-scattered plasmons at $\phi _{max} = \frac{\pi }{2}$ for NA = 1 and an ideal plasmon-diffraction-limited transverse (polar or in the $\phi$ direction) resolution of $\frac{{\lambda _{\mathrm {SP}}}}{2}$ for all points within the circle. Supplementary Figure S2 demonstrates these resolution effects. Depending on the specific application, a circular recording screen would likely be a more ideal geometry for in-line plasmonic DHM.

Practically, the resolution will be limited by the visibility of the finest resolvable fringes for plasmons diffracted at large angles. Since plasmons decay as they propagate, a linear recording geometry will be limited in size to roughly the propagation length of the plasmon. The circular recording geometry, however, can capture plasmons scattered at large angles before they decay. The hologram must also be sampled at a sufficient resolution to resolve the plasmonic fringes. With point-source geometry, the plasmonic fringes are magnified so that a high-resolution reconstruction can be created from a relatively lower-resolution hologram. Indeed, this magnification is necessary since it would not be possible to directly resolve unmagnified plasmonic fringes due to their shorter wavelengths. Exploiting this aspect could therefore allow plasmon-diffraction-limited in-plane imaging even with relatively lower-resolution microscope objectives.

As is typical with standard DHM and all focusing lenses limited by diffraction, the longitudinal resolution is not equal to the transverse resolution. As shown in the supplementary information, the longitudinal resolution $\Delta x$ of the reconstruction is given as:

7 $$\Delta x = \frac{{\lambda _{\mathrm {SP}}}}{{1 - \cos \phi _{\mathrm {max}}}}$$

This is also analogous to standard in-line DHM and represents the rough longitudinal extent of the point-spread function. This effect is also seen in the reconstructed images, e.g., in Fig. 3e, where point-like objects are elongated in the *x* direction. The effect is also seen in the circular recording geometry. Because it can capture all forward-scattered object waves, the effective NA of the circular recording geometry has been increased to NA = 1. However, as with the linear NSOM scans, the transverse (polar) resolution is still better than the longitudinal (radial) resolution because the weakly scattering objects are illuminated with radially outward plasmons and the reconstruction considers only radially inward back-propagation.

The full width of a single reconstructed point-source is indeed given by $\Delta x$ and $\Delta y$ as discussed above and shown in supplementary Figure S2. The full width also gives an estimate of the maximum spatial frequency that is resolvable in a plasmonic DHM reconstruction for a given NA, namely, $\sim \!1/\Delta x$ and $\sim \!1/\Delta y$. Two-point resolution in coherent imaging, however, will also depend on the relative phases of any point-sources: if the point-sources are in phase, two points will not be resolvable even if separated by $\sim \!\Delta x$ or $\sim \!\Delta y$; if the point-sources are out-of-phase, they will be resolvable even for separations slightly smaller than $\sim \!\Delta x$ or $\sim \!\Delta y$ and with higher contrast than an image formed with incoherent light. Supplementary Figure S3 demonstrates this effect. It is therefore challenging to describe a single resolution criterion^46^, especially for plasmonic DHM images that are composed of both longitudinal and transverse directions. The discussion given here has therefore focused on the approximate extent of the point-spread function as a resolution metric, which is appropriate for the sparse and weakly scattering objects, as is typical for in-line DHM.

**Fig. 6:**
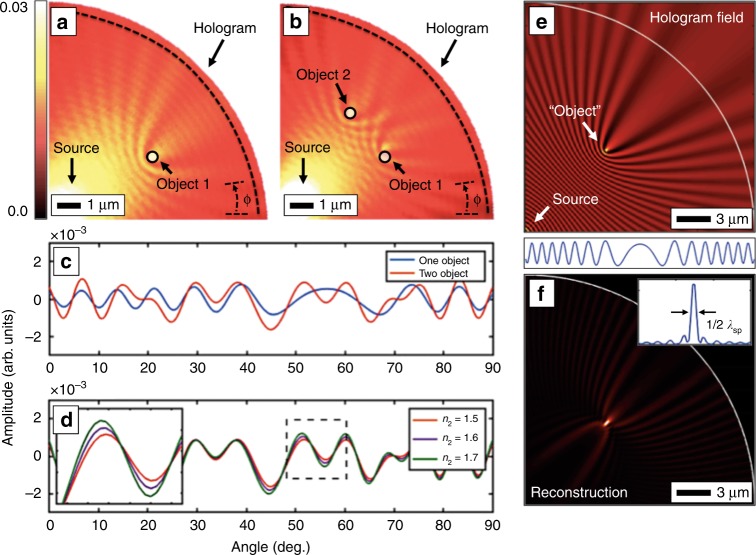
Simulations of plasmonic DHM. **a** Finite element simulations of a point-source interacting with either one or **b** two small dielectric objects and forming interference patterns. **c** The hologram is recorded on a semicircular screen and plotted vs. angle. The background pattern without any objects has been subtracted, leaving just the interference terms. **d** By changing the refractive index of one of the two objects, phase information is also accessible. **e** Analytical simulations of the interference between a point-plasmon reference and a point-plasmon object. The simulation covered the full circle, but only a portion is shown here. (below) Again, the hologram is extracted along a profile and the background is subtracted. **f** Digital reconstruction of the original point object from the hologram showing a plasmon-diffraction-limited spot at the focus

Finally, computer simulations were used to better understand the plasmonic hologram formation and its imaging potential. Full three-dimensional finite element simulations with COMSOL^TM^ as well as two-dimensional MATLAB^TM^ simulations of surface wave propagation were used. Figure 6 shows some of these results. Figure 6a shows a COMSOL simulation of a typical plasmonic DHM setup. These full simulations were necessarily smaller than the experimental holograms due to computer memory limitations. A quarter circle wedge with a 9-µm radius was used with perfectly match layer (PML) boundaries to absorb any outgoing radiation. A 660 nm Gaussian beam was focused onto a small 100 nm radius dot at the origin as in the experiments. This produced Hankel-type plasmons^42^ propagating radially outward as the reference plasmon wave. This wave interacted with one (Fig. 6a) or two (Fig. 6b) 200 nm diameter, 100 nm tall dielectric (*n* = 1.5) disks on the silver surface. The scattered plasmon wave, forming the object wave, interfered with the reference wave and produced the hologram. This standing wave interference pattern is clearly seen and is qualitatively similar to the NSOM scan shown in Fig. 2d. The hologram is a measure of the total plasmonic field intensity along the dashed line. In the simulations, it was possible to remove the object and record just the reference background separately. This background was then subtracted from the signal and the resulting hologram is plotted in Fig. 6c for both the single as well as the double object simulations. Naturally, the two object arrangements each produce two distinct holograms. Since holography is an interferometric technique, the refractive index of the objects is also recorded. Figure 6d shows a shift in the hologram fringes as one of the objects changes its refractive index by several small steps. Exploiting this shift could lead to novel forms of plasmonic biosensing or plasmonic phase imaging over an extended area. One advantage of our method for sensing applications is that a 2D array of sensing elements could be measured and analyzed with a single 1D hologram. In contrast to array-based, real-time plasmonic sensing that relies on capturing full 2D images with a CCD or CMOS camera limited to ~30 frames per second, plasmonic digital holography may provide for very fast acquisitions, i.e., microsecond time scales with a linear photodetector array to allow monitoring fast surface-binding processes and obtaining binding constants from kinetics parameters.

While finite element simulations provide full solutions to Maxwell’s equations, we also used MATLAB to explore the interference patterns between simpler arrangements, such as groups of point-sources. Figure 6e shows the standing wave field intensity between two-point-source Hankel-type waves, one at the origin as the reference and a weaker one as the object. The wavelength was set to 640 nm, i.e., the approximate surface-plasmon wavelength on a silver/air interface with 660 nm excitation, as stated above. The interference pattern between these two point-sources simulates the plasmonic DHM process, i.e., one point-source as the reference and one as the object, and produces qualitatively similar holograms (Fig. 6e) to the full COMSOL simulations as well as the experimental holograms shown above. The simulation covered the full circle, but only a portion is shown here. These holograms were then used to validate the reconstruction process and the resolution criteria. Taking the hologram from Fig. 6e and applying the propagation algorithms produces an image of the original point-source object at the correct location. Since the hologram was filtered to remove the background reference field, only the object is generated in the reconstruction (Fig. 6f). As shown in the inset, the transverse resolution is indeed limited by the wavelength of the plasmon to $\frac{{\lambda _{\mathrm {SP}}}}{2}$. Furthermore, as discussed above and shown in supplementary Figure S2, the resolution does not depend on the position of the point object within the circle, i.e., the NA is constant. Simulations also show that the “twin image” does provide some background interference. If the phase of the object wave were known completely, the reconstruction would yield a single point-source without the extraneous fringes. However, much effort has gone into reducing the effects of the twin image^47^. Indeed, various options would be directly and naturally applicable to plasmonic DHM, including iterative techniques^48^ and recording multiple holograms at two or more distances^49^. However, even without attempting to remove the twin image in this work, digital plasmonic reconstruction of the various test objects is still achieved.

To conclude, we have shown the plasmonic equivalent of in-line point-source DHM. A single-point-source plasmonic wave was used to illuminate various test objects, and the interference between the reference and the scattered object waves formed a hologram. The benefit of plasmonic DHM is the use of the relatively shorter wavelengths of SPs and their extreme sensitivity to surface features, objects, or defects. This hologram was accessed in two distinct ways, with either an NSOM system or using a fluorescent screen showing the general applicability of plasmonic DHM to a wide range of experiments. Digital reconstruction of these holograms reproduces the plasmonic field over an extended area, providing information about the various test objects. While these initial results demonstrate a proof-of-concept for plasmonic DHM, various improvements are anticipated. For example, several techniques should be directly applicable toward reduction of twin-image interference for higher-quality plasmonic image reconstruction^47^. Additionally, fast real-time imaging should also be possible using the fluorescent screens since only a small portion (i.e., a single line) of a single camera frame is necessary to extract the holograms and reconstruct the objects. While the limitations of in-line DHM are also present for in-line plasmonic DHM as presented here, i.e., imaging sparse and weakly scattering objects, there are various applications that could take advantage of such in-plane imaging. For example, a distribution of small sensing dots could be used as individual plasmonic sensing elements. It is challenging to fully decouple unknown shapes and unknown refractive index distributions from a single hologram, but changes in time of a single hologram for a known distribution of well-defined sensing elements would provide multiplex sensing capability. Interestingly, since the plasmonic holograms are imaged in our case via a microscope, the technique also captures “out-of-plane” plasmon-to-photon scattering in addition to “in-plane” plasmon-to-plasmon scattering at the same time, possibly enabling new studies of fundamental plasmon/nanoparticle interactions. Plasmonic DHM may therefore provide researchers with valuable new tools for nanoimaging, plasmonic phase imaging or interferometry, characterizing plasmonic devices, or novel forms of plasmonic biosensing.

## Materials and methods

The silver films were fabricated as follows. A silicon wafer chip was first cleaned with acetone, methanol, and isopropanol and then placed in a UV-ozone cleaner (Ossila) for 3 min. A 100-nm-thick film of silver was then deposited onto the silicon chip using a vacuum deposition chamber. A drop of optical adhesive (Norland 61) or Super Glue was placed onto the silver and adhered to a clean glass slide. After curing, the silver films were then peeled from the silicon wafer using a technique called “template stripping” to produce ultrasmooth (~1 nm RMS surface roughness as measured by the NSOM) silver surfaces with enhanced plasmonic properties^50^.

NSOM scans used a dual-probe instrument (Nanonics Multiview^TM^ 4000) where one probe was the excitation and the second the collection fiber. The illumination probe tip had an aperture of ~400 nm and was illuminated with a fiber-coupled 638 nm laser typically running with 10 mW power. The collection tip had an aperture of ~200 nm and was connected to an avalanche photodiode. The illumination tip was held in place, and the collection tip was then scanned across the surface to collect the plasmonic holograms. Typically, multiple line scans over the same area were averaged to obtain a better signal-to-noise ratio. To create the object samples, a 1000:1 dilute solution of polystyrene microspheres (either 200 or 400 nm nominal diameter) was mixed 1:1 with ethanol, and a small ~10 µL drop was placed onto a freshly stripped smooth silver chip and left to dry in air for several minutes. Various areas of the sample were then scanned. These line scans were then passed to a MATLAB script for hologram propagation, data analysis, and visualization. In this case, the plasmonic equivalent of the angular spectrum technique^18^ was used and computed using the FFT operation.

The second imaging technique used a fluorescent screen to record the hologram. A dye-doped (Oxazine 750, Exciton) fluorescent film of polymethylmethacrylate electron-beam resist (PMMA, Microchem) was created via spin coating (Ossila) onto a freshly stripped silver chip. The sample was then placed in a Hitachi SU1500 scanning electron microscope (SEM) equipped with a homemade LabVIEW^TM^ beam scanning and electron-beam lithography system. A typical sample consisted of a 25-µm-radius circle with a single dot in the center that, when illuminated with a focused laser, generated the source plasmon beam. In addition to the single central dot, each circle also had various arrangements of other small dots as the objects. The samples were then placed in an inverted microscope (Nikon) with a ×150 objective and imaged through a long-pass fluorescent filter (Semrock). The 660 nm laser illumination source (Laser Quantum) was typically set to 22 mW and entered the microscope through the back-illumination optics and was focused onto the small dot in the center of the circles. The plasmons propagating radially outward then entered the fluorescent PMMA area, allowing an EM CCD camera (Andor) to view the surface-plasmon intensity. These images were then passed to a MATLAB script for hologram propagation, data analysis, and visualization. In this case, the plasmonic equivalent of the helical spectrum technique^45^ was used and computed using the FFT operation.

## Electronic supplementary material

Supplementary Information: Digital Plasmonic Holography

## References

[1] Polman A (2008). Plasmonics applied. Science.

[2] Hecht B, Bielefeldt H, Novotny L, Inouye Y, Pohl DW (1996). Local excitation, scattering, and interference of surface plasmons. Phys. Rev. Lett..

[3] Lindquist NC, Nagpal P, McPeak KM, Norris DJ, Oh SH (2012). Engineering metallic nanostructures for plasmonics and nanophotonics. Rep. Prog. Phys..

[4] Gordon R, Sinton D, Kavanagh KL, Brolo AG (2008). A new generation of sensors based on extraordinary optical transmission. Acc. Chem. Res..

[5] Ditlbacher H, Krenn JR, Schider G, Leitner A, Aussenegg FR (2002). Two-dimensional optics with surface plasmon polaritons. Appl. Phys. Lett..

[6] Li L, Li T, Wang SM, Zhang C, Zhu SN (2011). Plasmonic Airy beam generated by in-plane diffraction. Phys. Rev. Lett..

[7] Hohenau A (2005). Dielectric optical elements for surface plasmons. Opt. Lett..

[8] Epstein I, Tsur Y, Arie A (2016). Surface-plasmon wavefront and spectral shaping by near-field holography. Laser Photonics Rev..

[9] Lee K, Lee SY, Jung J, Lee B (2015). Plasmonic achromatic doublet lens. Opt. Express.

[10] Fu YQ, Zhou XL (2010). Plasmonic lenses: a review. Plasmonics.

[11] Liu ZW (2005). Focusing surface plasmons with a plasmonic lens. Nano. Lett..

[12] Bezus EA, Doskolovich LL, Kazanskiy NL, Soifer VA, Kharitonov SI (2009). Design of diffractive lenses for focusing surface plasmons. J. Opt..

[13] Zentgraf, T., Liu, Y. M., Mikkelsen, M. H., Valentine, J. & Zhang, X. Plasmonic Luneburg and Eaton lenses. *Nat. Nanotechnol.* **6**, 151–155 (2011). 10.1038/nnano.2010.282 21258334

[14] Smolyaninov, I. I., Elliott, J., Zayats, A. V. & Davis, C. C. Far-field optical microscopy with a nanometer-scale resolution based on the in-plane image magnification by surface plasmon polaritons. *Phys. Rev. Lett.* **94**, 057401 (2005). 10.1103/PhysRevLett.94.057401 15783692

[15] Drezet A (2007). Surface plasmon polariton microscope with parabolic reflectors. Opt. Lett..

[16] Hohenau A (2011). Surface plasmon leakage radiation microscopy at the diffraction limit. Opt. Express.

[17] Brolo AG (2012). Plasmonics for future biosensors. Nat. Photonics.

[18] Kim MK (2010). Principles and techniques of digital holographic microscopy. SPIE Rev..

[19] Marquet P (2005). Digital holographic microscopy: a noninvasive contrast imaging technique allowing quantitative visualization of living cells with subwavelength axial accuracy. Opt. Lett..

[20] Schnars U, Jüptner WPO (2002). Digital recording and numerical reconstruction of holograms. Meas. Sci. Technol..

[21] Cuche E, Bevilacqua F, Depeursinge C (1999). Digital holography for quantitative phase-contrast imaging. Opt. Lett..

[22] Mann CJ, Yu LF, Lo CM, Kim MK (2005). High-resolution quantitative phase-contrast microscopy by digital holography. Opt. Express.

[23] Rappaz B (2005). Measurement of the integral refractive index and dynamic cell morphometry of living cells with digital holographic microscopy. Opt. Express.

[24] Yamaguchi I, Zhang T (1997). Phase-shifting digital holography. Opt. Lett..

[25] Cuche E, Marquet P, Depeursinge C (2000). Spatial filtering for zero-order and twin-image elimination in digital off-axis holography. Appl. Opt..

[26] Oh C, Isikman SO, Khademhosseinieh B, Ozcan A (2010). On-chip differential interference contrast microscopy using lensless digital holography. Opt. Express.

[27] Mudanyali O (2010). Compact, light-weight and cost-effective microscope based on lensless incoherent holography for telemedicine applications. Lab. Chip..

[28] Garcia-Sucerquia J (2006). Digital in-line holographic microscopy. Appl. Opt..

[29] Yu, X., Hong, J., Liu, C. G. & Kim, M. K. Review of digital holographic microscopy for three-dimensional profiling and tracking. *Opt. Eng.* **53**, 112306 (*2*014).

[30] Greenbaum A (2012). Imaging without lenses: achievements and remaining challenges of wide-field on-chip microscopy. Nat. Methods.

[31] Xu, W. B., Jericho, M. H., Meinertzhagen, I. A. & Kreuzer, H. J. Digital in-line holography for biological applications. *Proc. Natl Acad. Sci. USA* **98**, 11301–11305 (2001). 10.1073/pnas.191361398 PMC58724 11572982

[32] Jericho MH, Kreuzer HJ, Kanka M, Riesenberg R (2012). Quantitative phase and refractive index measurements with point-source digital in-line holographic microscopy. Appl. Opt..

[33] Choi YS, Lee SJ (2009). Three-dimensional volumetric measurement of red blood cell motion using digital holographic microscopy. Appl. Opt..

[34] Lim Y, Lee SY, Lee B (2011). Transflective digital holographic microscopy and its use for probing plasmonic light beaming. Opt. Express.

[35] Wei QS (2013). On-chip cytometry using plasmonic nanoparticle enhanced lensfree holography. Sci. Rep..

[36] Cetin AE (2014). Handheld high-throughput plasmonic biosensor using computational on-chip imaging. Light Sci. Appl..

[37] Zhang JW, Dai SQ, Ma CJ, Di JL, Zhao JL (2017). Common-path digital holographic microscopy for near-field phase imaging based on surface plasmon resonance. Appl. Opt..

[38] Gjonaj B (2013). Focusing and scanning microscopy with propagating surface plasmons. Phys. Rev. Lett..

[39] Archambault A, Teperik TV, Marquier F, Greffet JJ (2009). Surface plasmon Fourier optics. Phys. Rev. B.

[40] Kou SS (2016). On-chip photonic Fourier transform with surface plasmon polaritons. Light Sci. Appl..

[41] Huft PR, Kolbow JD, Thweatt JT, Lindquist NC (2017). Holographic plasmonic nanotweezers for dynamic trapping and manipulation. Nano. Lett..

[42] Nerkararyan S, Nerkararyan K, Janunts N, Pertsch T (2010). Generation of Hankel-type surface plasmon polaritons in the vicinity of a metallic nanohole. Phys. Rev. B.

[43] Johnson PB, Christy RW (1972). Optical constants of the noble metals. Phys. Rev. B.

[44] Teperik TV, Archambault A, Marquier F, Greffet JJ (2009). Huygens-Fresnel principle for surface plasmons. Opt. Express.

[45] Jackin BJ, Yatagai T (2010). Fast calculation method for computer-generated cylindrical hologram based on wave propagation in spectral domain. Opt. Express.

[46] Horstmeyer R, Heintzmann R, Popescu G, Waller L, Yang CH (2016). Standardizing the resolution claims for coherent microscopy. Nat. Photonics.

[47] Stoykova E, Kang H, Park J (2014). Twin-image problem in digital holography-a survey (invited paper). Chin. Opt. Lett..

[48] Latychevskaia, T. & Fink, H. W. Solution to the twin image problem in holography. *Phys. Rev. Lett.* **98**, 233901 (2007). 10.1103/PhysRevLett.98.233901 17677906

[49] Das, B. & Yelleswarapu, C. S. Dual plane in-line digital holographic microscopy. *Opt. Lett.* **35**, 3426–3428 (2010). 10.1364/OL.35.003426 20967088

[50] Nagpal P, Lindquist NC, Oh SH, Norris DJ (2009). Ultrasmooth patterned metals for plasmonics and metamaterials. Science.

