# Supplementary material for: Digital plasmonic holography
Source: Light Sci Appl. 2018 Aug 15;7:52. doi: 10.1038/s41377-018-0049-2 (PMC6107013; doi:10.1038/s41377-018-0049-2)
Supplement: Supplementary file 1 — Supplementary Information: Digital Plasmonic Holography [file 41377_2018_49_MOESM1_ESM.docx]

Supplementary Information: Digital Plasmonic Holography

*Joseph W. Nelson^1,+^, Greta R. Knefelkamp^1,+^, Alexandre G. Brolo^2,3,†^, Nathan C. Lindquist^1,*^*

^1^Department of Physics and Engineering, Bethel University, 3900 Bethel Drive, St Paul, MN 55112, USA

^2^Department of Chemistry, University of Victoria, 3800 Finnerty Road, Victoria, BC, V8P 5C2, Canada

^3^Center for Advanced Materials and Related Technologies (CAMTEC), University of Victoria, 3800 Finnerty Road, Victoria, BC, V8P 5C2, Canada

*^+^equal contribution*

^*^n-lindquist@bethel.edu

^†^agbrolo@uvic.ca

**Resolution Considerations.**

To determine the resolution of the plasmonic holograms, and adapting discussions from the digital holographic microscopy literature^1^, it is helpful to consider the interference pattern produced from a single point source reference and two objects a distance $D$ from the screen and separated by a small distance $\Delta y$. The screen has a full width of $S$ as shown in Figure S1a. The source and object plasmon waves can be represented by a cylindrical wave and the total field at the screen is given by:

$$U_{screen}=U_{0}\frac{e^{ik_{SP}r}}{\sqrt{r}}+U_{1}\frac{e^{ik_{SP}r_{1}}}{\sqrt{r_{1}}}+U_{2}\frac{e^{ik_{SP}r_{2}}}{\sqrt{r_{2}}}$$

Here $U_{0}$, $U_{1}$, $U_{2}$ are the amplitudes of the source and objects 1 and 2, respectively, and $k_{SP}=\frac{2\pi}{\lambda_{SP}}$ is the wavenumber of the surface plasmon. The intensity of the hologram has several terms:

$$\left| U_{screen} \right|^{2}=\frac{{U_{0}}^{2}}{r}+\frac{{U_{1}}^{2}}{r_{1}}+\frac{{U_{2}}^{2}}{r_{2}}+2 \frac{U_{1}U_{2}}{\sqrt{r_{1}r_{2}}}\cos\left( k_{SP}\left( r_{2}-r_{1} \right) \right)+\left\{ 2\frac{U_{0}U_{1}}{\sqrt{rr_{1}}}\cos\left( k_{SP}\left( r-r_{1} \right) \right)+2\frac{U_{0}U_{2}}{\sqrt{rr_{2}}}\cos\left( k_{SP}\left( r-r_{2} \right) \right) \right\}$$

The first $cos()$ term is the interference between the two point sources while the $cos()$ terms in braces are the holographic interference terms $H$. Note that if $U_{1}$,$U_{2}\ll U_{0}$ then many of these terms can be ignored and removed, as discussed in the main text, allowing experimental access to the holographic interference terms. For the purposes of this discussion, consider the situation where $U_{1}\approx U_{2}$, i.e. the two scattering objects are identical, and that $r_{1}\approx r_{2}$ since the separation distance $\Delta y$ is small. The holographic term can be written as:

$$H\approx4\frac{U_{0}U_{1}}{\sqrt{rr_{1}}}\cos\left( \frac{k_{SP}\left( 2r-r_{1}-r_{2} \right)}{2} \right)\cos\left( \frac{k_{SP}\left( r_{2}-r_{1} \right)}{2} \right)$$

The second $cos()$ term provides modulation relevant to the object spacing and the resolution of the hologram. To record this information, the contributions from the two point sources should be out-of-phase at the edge of the recording screen such that $k_{SP}\left( r_{2}-r_{1} \right)=\pi$. Since $\left( r_{2}-r_{1} \right)\approx\Delta y\sin\phi_{max}$, where $\phi_{max}$ is the maximum scattering angle, this means that the smallest resolvable distance $\Delta y$ is:

$$\Delta y=\frac{\lambda_{SP}}{2 \sin\phi_{max}}$$

which is identical to the classical diffraction limit for a lens that can capture light scattered at a maximum angle $\phi_{max}$. This is given as equation (6) in the main text and also represents the approximate transverse extent of the point-spread-function. Digital plasmonic holography therefore can achieve high-resolution in-plane imaging due to the short wavelengths of the plasmons. The effective numerical aperture NA for the linear holographic screen is NA = $\sin\phi_{max}= \frac{S/2}{\sqrt{{(\frac{S}{2})}^{2}+D^{2}}}$. For the circular recording geometry, it is possible to capture forward-scattered plasmons up to $\phi_{max}=\frac{\pi}{2}$ giving an effective NA = 1 with a resolution of $\Delta y=\frac{\lambda_{SP}}{2}$. This means that the transverse resolution will be constant for all points within the circle.

In the longitudinal or axial direction, i.e. along the direction of propagation, the calculation is similar. The geometry is shown in Figure S1b. The contribution from two point sources separated in the *x* direction is again given by the second $cos()$ term above and to properly record the information, it should generate an interference fringe across the entire screen. In this case, the maximum path-length-difference is $\Delta x$ and is at $\phi=0$ in the center of the screen. The resolution condition becomes $k_{SP}\left( \Delta x- \left( r_{2}-r_{1} \right) \right)=2\pi$. Since $\left( r_{2}-r_{1} \right)\approx\Delta x\cos\phi_{max}$, the smallest resolvable longitudinal distance $\Delta x$ is therefore:

$$\Delta x=\frac{\lambda_{SP}}{\left( 1-\cos\phi_{max} \right)}$$

which is also related to the classical diffraction-limited axial resolution. This is given as equation (7) in the main text and also represents the rough axial extent of the point-spread-function. For the circular recording geometry, the axial (or radial) resolution will therefore be $\Delta x=\lambda_{SP}$. Similar to before, this means that the radial resolution will be constant for all points within the circle. The behavior of these simple resolution equations for $\Delta x$ and $\Delta y$ are compared to simulations and are shown in Figure S2. Two closely spaced points are simulated in Figure S3.

**References.**

1. Garcia-Sucerquia, J., Xu, W., Jericho, S. K., Klages, P., Jericho, M. H., & Kreuzer, H. J., Digital in-line holographic microscopy. *Appl. Opt.* **45**, 836-850 (2006).

**Figures.**


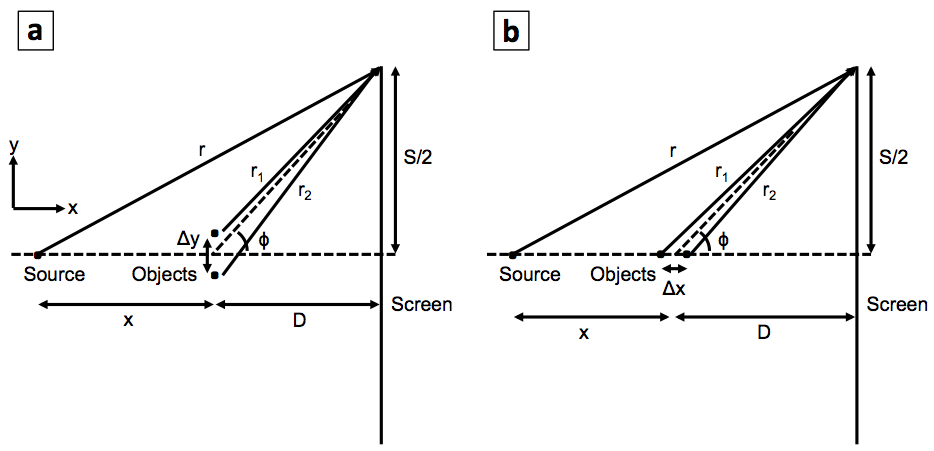


**Figure S1.** Geometry to calculate the hologram of two closely spaced points in (a) the transverse *y* direction and (b) the longitudinal *x* direction.


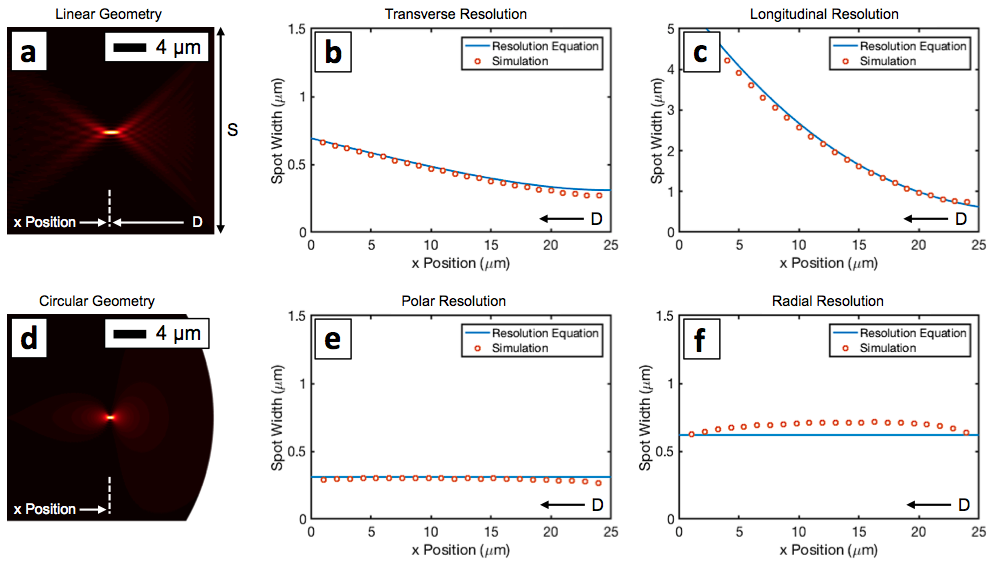


**Figure S2.** Comparison of the transverse and longitudinal resolution for the two recording geometries. The plasmon wavelength use here was $\lambda_{SP}=$620 nm. (a) A sample reconstruction of a single point object *D* = 12.5 µm from a linear recording screen of size *S* = 25 µm. For *D* = 12.5 µm the effective NA = sin(45°) ~ 0.71. The reconstruction is calculated from equation (3) as discussed in the main text. (b) Plot of the transverse resolution equation $\Delta y$ given above and the 1/*e* full width of the simulated reconstructed spot for various object-to-screen distances with the linear recording geometry. As the object becomes farther and farther from the screen (i.e., *D* increases and the *x* position decreases towards the source) the transverse spot size increases since the effective NA will decrease. The full transverse width of the simulated reconstructed spot agrees well with the resolution equation for $\Delta y$ derived above. (c) Plot of the longitudinal resolution equation $\Delta x$ given above and the 1/e full width of the reconstructed spot for various object-to-screen distances with the linear recording geometry. Again, the spot width increases with increasing distance *D* from the screen since the effective NA will decrease. (d) A sample reconstruction of a single point object *D* = 12.5 µm from a circular recording screen with a radius of 25 µm. The reconstruction is calculated from equation (4) as discussed in the main text. Due to the circular geometry, the effective NA is constant for all points within the circle. This is shown in (e) for the transverse, or polar, resolution and in (f) for the longitudinal, or radial, resolution. As with the linear screen simulations shown in (a)-(c), the simulated spot size matches the equations for $\Delta y$ and $\Delta x$ well.


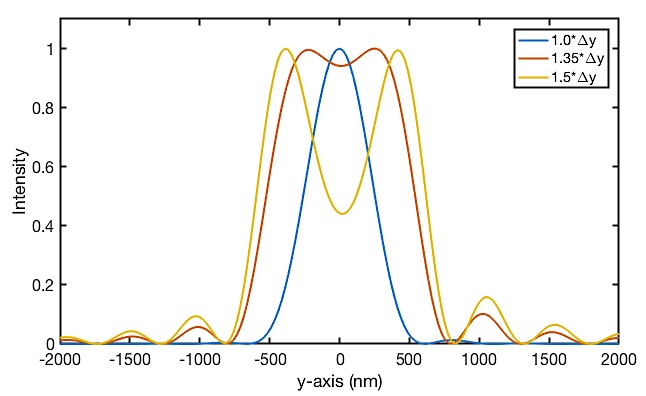


**Figure S3.** Two-point resolution simulations for a linear recording screen. The two point source objects are located a distance of D = 12.5 µm to a screen with a size of S = 25 µm. Similar to supplemental figure S2a, this corresponds to an effective NA of 0.71 and a lateral resolution of $\Delta y$ of 450 nm for a plasmon wavelength of 640 nm. The source is 25 µm from the screen. The plot shows cross sections of normalized reconstructed fields at the location of two point sources separated by a lateral distance of $1.0*\Delta y$, $1.35*\Delta y$, and $1.5*\Delta y$. While the size of a single point source is given by $\Delta y$, here the two point sources are both excited in phase, meaning that the actual resolvable distance is slightly larger than $\Delta y$ as discussed in the main text.
